# Triple negative breast cancer subtypes and pathologic complete response rate to neoadjuvant chemotherapy

**DOI:** 10.18632/oncotarget.25413

**Published:** 2018-05-29

**Authors:** Angela Santonja, Alfonso Sánchez-Muñoz, Ana Lluch, Maria Rosario Chica-Parrado, Joan Albanell, José Ignacio Chacón, Silvia Antolín, José Manuel Jerez, Juan de la Haba, Vanessa de Luque, Cristina Elisabeth Fernández-De Sousa, Luis Vicioso, Yéssica Plata, César Luis Ramírez-Tortosa, Martina Álvarez, Casilda Llácer, Irene Zarcos-Pedrinaci, Eva Carrasco, Rosalía Caballero, Miguel Martín, Emilio Alba

**Affiliations:** ^1^ Instituto de Investigación Biomédica de Málaga (IBIMA), Hospitales Universitarios Regional y Virgen de la Victoria, Málaga, Spain; ^2^ Laboratorio de Biología Molecular del Cáncer, Centro de Investigaciones Médico-Sanitarias (CIMES), Universidad de Málaga, Málaga, Spain; ^3^ Unidad de Gestión Clínica Intercentro de Oncología, Instituto de Investigación Biomédica de Málaga (IBIMA), Hospitales Universitarios Regional y Virgen de la Victoria, Málaga, Spain; ^4^ Centro de Investigación Biomédica en Red de Oncología, CIBERONC-ISCIII, Madrid, Spain; ^5^ Spanish Breast Cancer Research Group (GEICAM), Madrid, Spain; ^6^ Department of Oncology and Hematology, Hospital Clínico Universitario, Valencia, Spain; ^7^ INCLIVA Biomedical Research Institute, Universidad de Valencia, Valencia, Spain; ^8^ Cancer Research Program, IMIM (Hospital del Mar Medical Research Institute), Medical Oncology Service, Hospital del Mar, Barcelona, Spain; ^9^ Universitat Pompeu Fabra, Barcelona, Spain; ^10^ Medical Oncology Service, Hospital Virgen de la Salud, Toledo, Spain; ^11^ Medical Oncology Service, Complejo Hospitalario Universitario de A Coruña, La Coruña, Spain; ^12^ Department of Languages and Computer Science, Instituto de Investigación Biomédica de Málaga (IBIMA), Universidad de Málaga, Málaga, Spain; ^13^ Medical Oncology Service, Complejo Hospitalario Reina Sofía, Córdoba, Spain; ^14^ The Maimonides Institute for Biomedical Research (IMIBIC), Córdoba, Spain; ^15^ Department of Pathology, Hospitales Universitarios Regional y Virgen de la Victoria, Málaga, Spain; ^16^ Department of Pathology, Faculty of Medicine, Universidad de Málaga, Málaga, Spain; ^17^ Department of Oncology, Complejo Hospitalario de Jaén, Jaén, Spain; ^18^ Department of Pathology, Complejo Hospitalario de Jaén, Jaén, Spain; ^19^ Medical Oncology Service, Hospital Costa del Sol, Marbella, Málaga, Spain; ^20^ Health Services Research on Chronic Diseases Network – REDISSEC, Marbella, Málaga, Spain; ^21^ Instituto de Investigación Sanitaria Gregorio Marañón, Universidad Complutense de Madrid, Madrid, Spain

**Keywords:** triple negative breast cancer, subtyping, pathologic complete response, neoadjuvant therapy, carboplatin

## Abstract

Triple negative breast cancer (TNBC) is a heterogeneous disease with distinct molecular subtypes that differentially respond to chemotherapy and targeted agents. The purpose of this study is to explore the clinical relevance of Lehmann TNBC subtypes by identifying any differences in response to neoadjuvant chemotherapy among them. We determined Lehmann subtypes by gene expression profiling in paraffined pre-treatment tumor biopsies from 125 TNBC patients treated with neoadjuvant anthracyclines and/or taxanes +/- carboplatin. We explored the clinicopathological characteristics of Lehmann subtypes and their association with the pathologic complete response (pCR) to different treatments. The global pCR rate was 37%, and it was unevenly distributed within Lehmann’s subtypes. Basal-like 1 (BL1) tumors exhibited the highest pCR to carboplatin containing regimens (80% vs 23%, p=0.027) and were the most proliferative (Ki-67>50% of 88.2% vs. 63.7%, p=0.02). Luminal-androgen receptor (LAR) patients achieved the lowest pCR to all treatments (14.3% vs 42.7%, p=0.045 when excluding mesenchymal stem-like (MSL) samples) and were the group with the lowest proliferation (Ki-67≤50% of 71% vs 27%, p=0.002). In our cohort, only tumors with LAR phenotype presented non-basal-like intrinsic subtypes (HER2-enriched and luminal A). TNBC patients present tumors with a high genetic diversity ranging from highly proliferative tumors, likely responsive to platinum-based therapies, to a subset of chemoresistant tumors with low proliferation and luminal characteristics.

## INTRODUCTION

Triple negative breast cancer (TNBC) includes a heterogeneous subgroup of tumors accounting for approximately 15-20% of all breast cancers. TNBC is clinically defined by the absence of expression of estrogen receptor (ER), progesterone receptor (PR), and human epidermal growth factor receptor 2 (HER2) amplification/overexpression. TNBC presents a more aggressive natural history and worse disease-specific outcomes than other breast cancer subtypes. Anthracycline and taxane-based chemotherapy has been traditionally the mainstay of therapy for TNBC patients. Nevertheless, platinum-based chemotherapy, with a DNA-damaging mechanism of action, has been incorporated into the neoadjuvant and metastatic settings. Patients with TNBC do not benefit from targeted therapies such as endocrine therapy or trastuzumab, and no appropriate molecular targets have been identified yet [1, 2].

Neoadjuvant chemotherapy has been historically used to downstage unresectable tumors for better loco-regional control and higher conservative surgery rate. The neoadjuvant approach also represents an excellent *in vivo* test of tumors´ biological sensitivity and of drugs´ clinical efficacy. Therefore, it facilitates cancer research and works towards personalized medicine [3, 4]. After receiving neoadjuvant chemotherapy, about 30% of TNBC patients present a complete absence of residual invasive tumor or pathologic complete response (pCR). Achieving a pCR improves these patients´ prognoses to the point that their disease-free survival and overall survival are similar to patients with less aggressive tumors. However, TNBC patients with residual disease after chemotherapy have worse survival and prognosis than those non-triple negative [5-7]. In TNBC, pCR is therefore considered a potential surrogate marker for survival [7].

Gene expression analysis studies have contributed to unveil TNBC heterogeneity by demonstrating that it is composed of all the intrinsic subtypes, being basal-like the most common subtype (70%) [8]. Thus, not all triple negative are basal-like by gene expression and not all basal-like are triple negative by immunohistochemistry. Recently, Lehmann and colleagues performed a more thorough dissection of TNBC into 7 distinct subtypes based on gene expression profiling. This classification included 6 stable subtypes consisting in: two basal-like (BL1 and BL2), an immunomodulatory (IM), a mesenchymal (M), a mesenchymal stem-like (MSL), and a luminal androgen receptor (LAR) subtype; and an unstable (UNS) subtype. These subtypes were reproduced and pharmacologically targeted in breast cancer cell lines as proof of concept that they can inform therapy selection [9]. However, the clinical relevance of the subtyping defined by Lehmann et al. is still unclear and more research is needed to clarify its impact on TNBC treatment decisions.

We believe there is a need for proper validation of the value of TNBC subtyping regarding response to treatment and survival outcome. Thus, a first highly valuable step would be testing how Lehmann´s classification predicts tumor pCR in the neoadjuvant setting. The main aim of this study was to assess the clinical relevance of these 7 molecular subtypes by ascertaining their correlation with pCR, as a surrogate marker for overall survival, in a cohort of 125 TNBC patients treated with anthracyclines and/or taxanes +/- carboplatin in the neoadjuvant setting. In addition, we evaluated the activity of platinum salts in BL subtypes, given that drugs with DNA-damaging mechanisms of action have proven to be effective in tumors with DNA repair defects that characterize these subtypes [9].

## RESULTS

### Characteristics of the TNBC population

Patients’ clinicopathological characteristics in the global population (N=125) are shown in Table 1. Patients were mainly pre-menopausal (56%), with positive lymph nodes (59%), high histological grade (63% had grade 3) and high proliferation (median Ki-67 index of 65%, range 5-100%). Close to a third of the patients (27%) received carboplatin in the global population, being this percentage substantially higher in patients from the GEICAM/2006-03 clinical trial (56%). The pCR rate in the global population was 37% although it was unevenly distributed across Lehmann subtypes.

**Table 1:** Patients’ characteristics

Characteristics	N (%)
Age at diagnosis (years)	
Median	48
Range	29-76
Tumor size (cm)	
<2	9 (7.2%)
2-5	90 (72%)
>5	24 (19.2%)
NA	2 (1.6%)
Lymph node status	
N0	38 (30.4%)
N+	74 (59.2%)
NA	13 (10.4%)
Histological grade	
1	3 (2.4%)
2	28 (22.4%)
3	79 (63.2%)
NA	15 (12%)
Ki-67 (%)	
≤50	39 (31.2%)
>50	83 (66.4%)
NA	3 (2.4%)
Intrinsic subtypes	
Basal	104 (83.2%)
Non-basal	6 (4.8%)
NA	15 (12%)
Lehmann subtypes	
BL1	17 (13.6%)
BL2	15 (12%)
M	22 (17.6%)
MSL	9 (7.2%)
IM	25 (20%)
LAR	14 (11.2%)
UNS	17 (13.6%)
NA	6 (4.8%)
Treatment	
A and/or T	91 (72.8%)
A+T+Cb	34 (27.2%)
pCR	
Yes	46 (36.8%)
No	79 (63.2%)

Abbreviations: N0, No node involvement; N+, node involvement; NA, not available; A, anthracyclines; T, taxanes; Cb, carboplatin.

### TNBC subtyping and clinicopathological variables

#### Lehmann subtypes

Of the 125 TNBC patients with evaluable pathologic response after neoadjuvant chemotherapy, RNA with sufficient quantity and quality for Lehmann subtyping was collected from 119 (95%) of them. Of these, 102 (86%) were classified as stable and 17 (14%) as unstable (UNS) (Table 1). A more detailed description of the clinicopathological variables in every Lehmann subtype is shown in Table 2. Ki-67 index (dichotomized using a cut-off >50%) was associated with Lehmann subtyping (p=0.002). BL1 samples had the highest proliferation rates (88.2% of BL1 patients vs. 63.7% of patients with other subtypes had Ki-67>50%, p=0.02) and LAR the lowest (71% of LAR patients vs. 27% of patients with other subtypes had Ki-67≤50%, p=0.002).

**Table 2:** Patients’ characteristics by Lehmann subtype, N (%) and p-values of the comparison between all subtypes and all subtypes except MSL

Characteristics	BL1(N=17)	BL2(N=15)	M(N=22)	MSL(N=9)	IM(N=25)	LAR(N=14)	UNS(N=17)	p all subtypes	p excluding MSL
Age (years)									
<50	10 (58.8%)	9 (60%)	12 (54.5%)	3 (33.3%)	20 (80%)	6 (42.9%)	10 (58.8%)		
≥50	7 (41.2%)	6 (40%)	10 (45.5%)	6 (66.7%)	5 (20%)	8 (57.1%)	7 (41.2%)	0.1791	0.2686
Tumor size (cm)									
<2	3 (17.6%)	0	3 (13.6%)	0	2 (8%)	0	1 (5.9%)		
2-5	13 (76.5%)	13 (86.7%)	16 (72.7%)	7 (77.8%)	17 (68%)	9 (64.3%)	11 (64.7%)		
>5	1 (5.9%)	2 (13.3%)	2 (9.1%)	2 (22.2%)	6 (24%)	4 (28.6%)	5 (29.4%)	0.5033	0.3979
NA	0	0	1 (4.6%)	0	0	1 (7.1%)	0		
Lymph node status									
N0	8 (47.1%)	4 (26.7%)	5 (22.7%)	4 (44.4%)	7 (28%)	3 (21.4%)	6 (35.3%)		
N+	8 (47.1%)	8 (53.3%)	12 (54.6%)	5 (55.6%)	16 (64%)	10 (71.4%)	10 (58.8%)		
NA	1 (5.8%)	3 (20%)	5 (22.7%)	0	2 (8%)	1 (7.2%)	1 (5.9%)	0.7903	0.7449
Histological grade									
1	0	1 (6.7%)	0	0	0	1 (7.1%)	1 (5.9%)		
2	5 (29.4%)	0	7 (31.8%)	2 (22.2%)	5 (20%)	5 (35.7%)	3 (17.6%)		
3	10 (58.8%)	13 (86.6%)	11 (50%)	4 (44.5%)	18 (72%)	7 (50.1%)	11 (64.7%)		
NA	2 (11.8%)	1 (6.7%)	4 (18.2%)	3 (33.3%)	2 (8%)	1 (7.1%)	2 (11.8%)	0.1438	0.0912
Ki-67 (%)									
≤50	1 (5.9%)	4 (26.7%)	9 (40.9%)	5 (55.6%)	4 (16%)	10 (71.4%)	5 (29.4%)		
>50	15 (88.2%)	11 (73.3%)	13 (59.1%)	4 (44.4%)	21 (84%)	4 (28.6%)	12 (70.6%)		
NA	1 (5.9%)	0	0	0	0	0	0	0.0015^*^	<0.001^*^
Intrinsic subtypes									
basal	16 (94.1%)	15 (100%)	20 (90.9%)	8 (88.9%)	25 (100%)	5 (35.7%)	12 (70.6%)		
non-basal	0	0	0	0	0	5 (35.7%)	1 (5.9%)		
NA	1 (5.9%)	0	2 (9.1%)	1 (11.1%)	0	4 (28.6%)	4 (23.5%)	<0.001^*^	<0.001^*^
Treatment									
A and/or T	12 (70.6%)	12 (80%)	18 (81.8%)	7 (77.8%)	17 (68%)	10 (71.4%)	12 (70.6%)		
A+T+Cb	5 (29.4%)	3 (20%)	4 (18.2%)	2 (22.2%)	8 (32%)	4 (28.6%)	5 (29.4%)	0.9447	0.8916
pCR									
Yes	8 (47.1%)	7 (46.7%)	9 (40.9%)	3 (33.3%)	10 (40%)	2 (14.3%)	7 (41.2%)		
No	9 (52.9%)	8 (53.3%)	13 (59.1%)	6 (66.7%)	15 (60%)	12 (85.7%)	10 (58.8%)	0.5714	0.4539

Abbreviations: NA, not available; N0, No node involvement, N+, node involvement. NA, not available A, anthracyclines; T, taxanes; Cb, carboplatin.

^*^p≤0.05.

#### Intrinsic subtypes

We further classified 110 (88%) of the tumor samples into intrinsic subtypes. Of these, 104 (94.5%) were classified as basal-like and 6 (5.4%) as non-basal-like (Table 1). Non-basal-like samples included 5 HER2-enriched and 1 luminal A; due to this low number we combined these 6 samples into a group of non-basal-like samples for further analyses. As highlighted in the methodology, 36% of the tumors in this study were collected as part of the GEICAM/2006-03 trial, which eligibility criteria included the *core basal* definition, a more restrictive triple negative definition (triple negative status plus EGFR-positive and/or CK5/6-positive). The fact that all these samples were already basal by immunohistochemistry could have caused an overrepresentation of basal-like tumors compared to the triple negative-only definition. However, we observed a comparable proportion of samples classified as basal-like when excluding this subset of *core basal* samples defined by immunohistochemistry (94.5% of basal-like samples in the global cohort vs. 92.2% in the triple negative-only cohort, p=0.56).

#### Correlation between Lehmann and intrinsic subtyping

**Figure 1:**
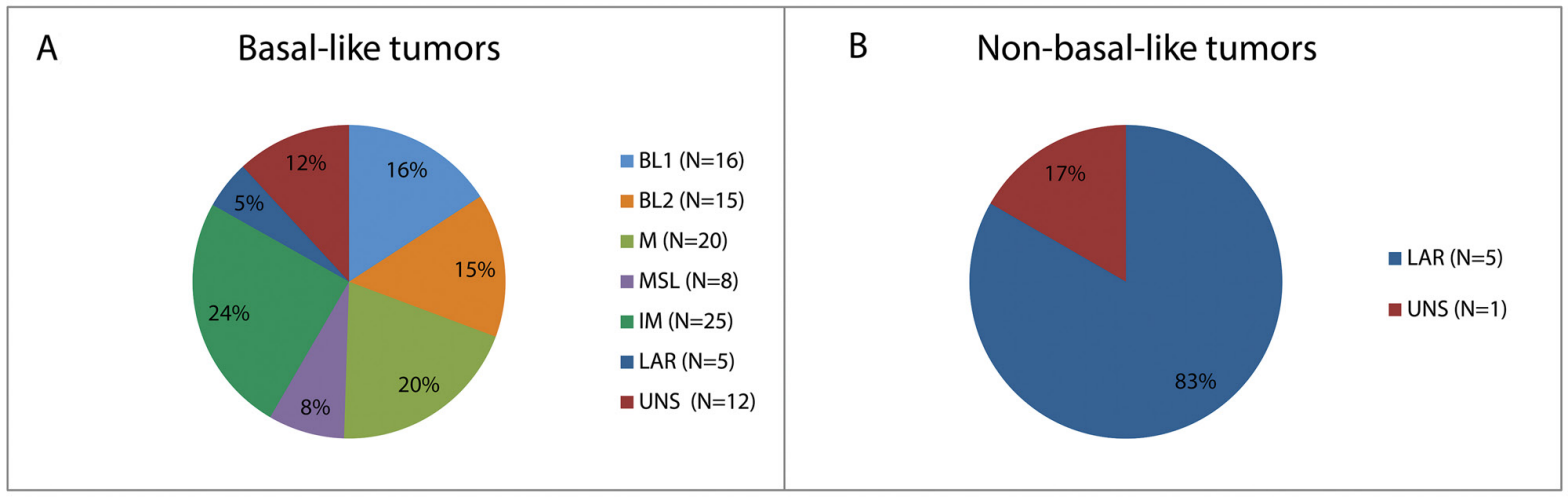
Distribution of Lehmann subtypes within intrinsic subtypes **(A)** Distribution of Lehmann subtypes in molecular basal-like tumors. **(B)** Distribution of Lehmann subtypes in molecular non-basal-like tumors.

We found a strong concordance between Lehmann and intrinsic subtyping (p<0.001, Table 2) mainly because the only stable group with non-basal-like samples is the LAR subtype. The distribution of Lehmann subtypes into basal-like and non-basal-like intrinsic subtypes is shown in Figure 1. Of the 5 LAR samples classified as non-basal-like, 4 were HER2-enriched and 1 was luminal A. We had enough tumor tissue to successfully determine the expression of the androgen receptor (AR) in the luminal A and three out of four HER2-enriched tumors; all overexpressed AR and were histopathologically consistent with apocrine carcinomas.

### Pathological complete response to different neoadjuvant treatments

We first analyzed the association between the different clinicopathological variables and the pathological complete response (pCR) to neoadjuvant treatment in the global sample. High expression of Ki-67 (using a cut-off >50%, p=0.037) and bigger clinical tumor size (≤2cm vs. >2cm, p=0.024) were associated with higher number of pCRs achieved. Clinical tumor size was the only variable that remained associated with pCR when performing a multivariate analysis (p=0.002).

### pCR to overall neoadjuvant treatment by Lehmann subtype

**Figure 2:**
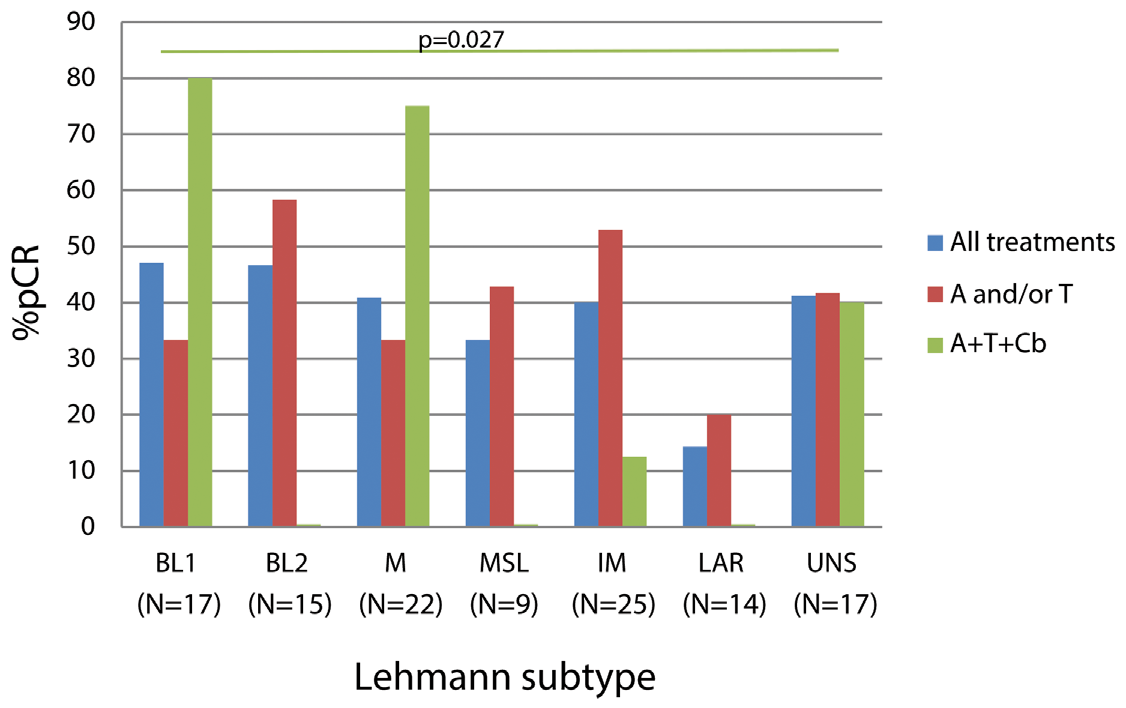
Percentage of pCR associated to the different Lehmann subtypes by treatment The green horizontal line represents the comparison of the percentage of pCR to sequential anthracyclines and taxanes plus carboplatin of BL1 versus the rest of patients and its associated p-value. The number of patients receiving every treatment within each Lehmann subtype can be found at Table 2. Abbreviations: A, anthracyclines; T, taxanes; Cb, carboplatin.

Next, we analyzed the pCR rates achieved by Lehmann subtype (see Figure 2). As recently Prat and colleagues highlighted that Lehmann subtyping ignores TNBC samples that are highly contaminated with normal breast tissue, which are mostly classified as MSL [10], we performed all analyses with and without the MSL group to avoid missing relevant associations between clinicopathological variables and Lehmann subtypes.

We found no statistically significant association between Lehmann subtypes and pCR to overall neoadjuvant treatment (p=0.571) in spite of the wide range of pCR observed (from 47.1% in BL1 to 14.3% in LAR). LAR patients were the most chemoresistant (14.3% of pCR in LAR vs. 41.9% in the remaining subtypes combined, p=0.077) and when excluding MSL samples, this difference in response appeared to be more pronounced (14.3% of pCR in LAR patients vs. 42.7% in the remaining groups except MSL, p=0.046).

### pCR to the different treatments in overall sample and by Lehmann subtype

We found no differences in pCR rates in the global population (without subtyping) when comparing patients treated with and without carboplatin (40.9% vs. 32.3% of pCR, respectively; p=0.521).

When analyzing the pCR response to the different treatments received by Lehmann subtype, we did not observe a difference in pCR rates to standard chemotherapy (sequential anthracyclines and/or taxanes) by Lehmann subtypes (p=0.556). When comparing the pCR rates in patients treated with carboplatin in each of the Lehmann subtypes, we observed that patients with BL1 tumors were the most benefited of the addition of carboplatin (80% of pCR in BL1 patients vs. 23% of pCR in the remaining groups, p=0.027).

## DISCUSSION

Triple negative breast cancer (TNBC) is a commonly used umbrella term for a histologic group of tumors which, from a molecular perspective, are vastly heterogeneous. In fact, TNBC includes a wide range of entities differing in biology and response to chemotherapy and targeted therapies, and, thus, leading to different clinical outcomes. Recently, several TNBC classifications have been published illustrating the existing inconsistencies both in the definition of disease subgroups and of their corresponding clinical outcomes [9, 11-13]; only the subtypes termed as LAR appear to be consistent across all the studies, though it is unclear whether these classifications are predictive of treatment efficacy.

In this report, we analyzed a combined dataset of TNBC patients treated with anthracyclines and/or taxanes +/- carboplatin in the neoadjuvant setting. First, we classified them into the TNBC Lehmann subtypes and evaluated their clinicopathological characteristics and, second, we explored the chemosensitivity of these subtypes to the different neoadjuvant treatments administrated. To our knowledge, this is the first study evaluating the prognostic role of Lehmann TNBC subtypes in the neoadjuvant setting of patients treated with and without platinum salts.

Based on our results, LAR is the least proliferative tumor subtype and the most chemoresistant one. Despite its significantly lower response to neoadjuvant chemotherapy in comparison to the other subtypes, LAR has been associated with a favorable prognosis when defined by immunohistochemistry as AR-positive [14-16]. This may be, in part, because LAR is the only subtype including non-basal-like tumors [17, 18] which could explain the low proliferation and pCR rates observed in our study. However, there are controversial results in the literature when LAR is defined by gene-expression, having the best [19, 20] or worse prognosis [9, 21] within TNBC and when analyzing all breast cancer subtypes [22]. As expected, our LAR samples expressed AR and were histologically consistent with apocrine carcinomas [23]. Recently published early phase II clinical trials results suggest that antiandrogen therapy may target the AR-positive subset of TNBCs [24-26].

In our study, BL1 subtype appeared to be particularly sensitive to chemotherapy regimens including a platinum agent. This is of major significance because in the past few decades there has been considerable interest in platinum salts as treatment for TNBC given that homologous recombination deficiency (HRD) sensitizes tumor cells to these agents inducing cell death. Although, results from phase II studies involving unselected TNBC patients in the neoadjuvant setting have been conflicting [27-29], TNBC tumors harboring a high HRD score seem to benefit from platinum-based therapy [30].

The results of this study should be interpreted in the context of its limitations. First, the actual number of samples analyzed under each Lehmann subtype is limiting; second, we used formalin-fixed paraffin-embedded (FFPE) samples for gene expression analysis, which could present differences when compared to analysis performed in fresh/frozen tissue; and, third, patients did not receive homogeneous neoadjuvant treatments, although all patients did receive anthracyclines and/or taxanes +/- carboplatin regimes.

In conclusion, our results confirm the high genetic diversity within TNBC tumors, although, rather than falling into discreet categories, TNBC disease may be considered a spectrum of tumors with varying clinically-relevant characteristics. On one extreme of this spectrum, we would have BL1 tumors, a highly proliferative subtype with its likely deficiencies in HRD resulting in a high pCR to platinum salts-based therapies. On the other side, we would find LAR, a tumor subtype characterized by a low proliferation and a low response to standard chemotherapy. In between, we would find patients with TNBC tumors that cannot be classified into any subgroup further than the standard definition of TNBC. These results may have important implications in the design, implementation, and evaluation of future clinical trials aimed at further exploration of the clinical utility of TNBC subtyping.

## MATERIALS AND METHODS

### Patients and samples

We performed a retrospective analysis on 125 patients with invasive TNBC including 45 (36%) from a randomized phase II trial (GEICAM/2006-03, ClinicalTrials.gov: NCT00432172) with a prospective collection of FFPE tumor samples and associated clinical data, and 80 (64%) patients for whom we had retrospectively collected FFPE tumor samples and data from four collaborating hospitals. GEICAM/2006-03 study was the first to investigate whether adding carboplatin to one of the most commonly used standard chemotherapy combinations (neoadjuvant epirubicin/cyclophosphamide followed by docetaxel) increased the pCR rate in basal-like breast cancer patients. Further information about this trial can be found elsewhere [27].

To be included in this retrospective study, patients had to be over 18-years old females with histologically confirmed invasive TNBC (ER-negative, PR-negative, HER2-negative). All patients received standard neoadjuvant chemotherapy consisting of anthracyclines and/or taxanes with or without carboplatin. Patients included in the study had already undergone surgery during which data on their pathological response was assessed and collected in every collaborating hospital from the restrospective cohort and centrally in the GEICAM cohort. pCR was defined as the absence of invasive carcinoma in the breast and lymph nodes according to the Miller & Payne criteria [31]. Samples were managed and/or provided by the Málaga Hospital-IBIMA Biobank and GEICAM Biobank. All patients participating in the study provided written informed consent and study protocols were approved by the corresponding institutional ethical committees.

### Samples processing

#### Immunohistochemistry

Analyses were performed with FFPE biopsies obtained before neoadjuvant treatment. Immunostaining was performed and assessed in every collaborating hospital using estrogen receptor (ER, Clone SP1), progesterone receptor (PR, Clone Y85), cytokeratin 5/6 (CK5/6, Clone D5/16B4), Ki-67 (Clone SP6) (Master Diagnóstica, Granada, Spain), epidermal growth factor receptor (EGFR, Clone 2-18C9), androgen receptor (AR, Clone AR441), and human epidermal growth factor receptor 2 (HER2, HercepTest) (DakoCytomation, Glostrup, Denmark). Samples from all patients were defined as triple negative by immunohistochemistry as ER-negative, PR-negative, and HER2-negative. AR status was assessed only in the samples classified as non-basal-like by PAM50 in order to evaluate if they were compatible with apocrine carcinomas. ER, PR and AR status were considered negative if <1% of cells stained positively [32]. HER2 status was considered negative if either immunohistochemical results were 0 to +1, or were +2 and FISH results were negative [33]. All GEICAM/2006-03 samples were CK5/6-positive and/or EGFR-positive by immunohistochemistry and therefore defined as *core basal* [34]. CK5/6 and EGFR tumor immunoreactivity was determined only in the GEICAM cohort and categorized as negative (immunohistochemical score 0), and positive (immunohistochemical score 1-3). Information about Ki-67 was collected as a continuous variable as well as a dichotomized variable defined by a cut-off point of 50% [35].

#### RNA extraction

Hematoxylin and eosin staining of a slide-mounted tumor section were reviewed, by independent pathologists for the GEICAM and non-GEICAM collections, to identify the area of invasive breast carcinoma. Tumor area was manually macrodissected to obtain an enriched tumor RNA. Extraction and purification was performed with the RNeasy FFPE Kit (Qiagen) from 3-6 sections of 10μm and quality assessment was conducted using a 2100 Bioanalyzer (Agilent Technologies) and a Nanodrop spectrophotometer (Thermo Scientific).

#### TNBC subtyping

Gene expression analyses to classify samples into 1 of the 4 intrinsic subtypes (luminal A, luminal B, HER2-enriched, basal-like) were performed on an nCounter Analysis System (NanoString Technologies). For further analyses, we grouped samples as basal-like and non-basal-like (luminal A, luminal B and HER2-enriched); as the majority of triple negative breast tumors are basal-like we expected a low number of the remaining subtypes. Samples from the GEICAM/2006-03 clinical trial were profiled using the PAM50 classifier and analyzed by means of a clinical algorithm for subtype prediction [36], discarding samples classified as normal-like. The retrospective collection of patients was classified according the Prosigna assay [37], which includes a proprietary algorithm based on the PAM50 gene signature [38].

RNA processing and microarray analysis performed to classify the samples into Lehmann TNBC subtypes were performed at the Microarray Analysis Service (SAM) core facility from Hospital del Mar Medical Research Institute (IMIM), using exclusively Affymetrix Technology (Affymetrix, Santa Clara, CA). RNA was amplified and labelled using the SensationPlus™ FFPE Amplification and WT Labeling kit. The resulting cDNA was hybridized to the GeneChip® Human Transcriptome Array 2.0. Data were normalized with the Robust Multichip Analysis (RMA) algorithm in the Affymetrix Expression Console (EC, v.1.4.1). Data were annotated in the statistical computing environment R (v.3.2.3) using hg19 human genome built and duplicated genes were mean summarized. Gene expression data are available in the GEO repository (accession GSE106977), at https://www.ncbi.nlm.nih.gov/geo/query/acc.cgi?acc=GSE106977. Subtypes were identified with the web-based tool TNBCtype [39].

### Statistical analyses

Pearson’s chi-square test was used to perform contingency table and goodness-of-fit tests, and Fisher’s exact test when any of expected values in cells were less than 5. Student’s *t*-test was also used to test the null hypothesis under the assumption of the two populations having equal means. A logistic regression multivariate analysis was performed, using a stepwise forward and backward selection procedure to select the most important variables of the model based on the Akaike information criterion (AIC). All statistical analyses were conducted in the statistical computing environment R (v. 3.3.1). Because the Lehmann’s MSL subtype can have an overrerpresentation of normal breast tissue [10], we performed all analysis both including and excluding the MSL subtype to avoid missing relevant associations.

## Abbreviations

AR: Androgen receptor
BL1: Basal-like 1
BL2: Basal-like 2
CK5/6: Cytokeratin 5/6
EGFR: Epidermal growth factor receptor
ER: Estrogen receptor
FFPE: Formalin-fixed paraffin-embedded
HER2: Human epidermal growth factor receptor 2
HRD: Homologous recombination deficiency
IM: Immunomodulatory
LAR: Luminal-androgen receptor
M: Mesenchymal
MSL: Mesenchymal stem-like
pCR: Pathological complete response
PR: Progesterone receptor
TNBC: Triple negative breast cancer
UNS: Unstable
